# Optical coherence tomography-based deep-learning model for detecting central serous chorioretinopathy

**DOI:** 10.1038/s41598-020-75816-w

**Published:** 2020-11-02

**Authors:** Jeewoo Yoon, Jinyoung Han, Ji In Park, Joon Seo Hwang, Jeong Mo Han, Joonhong Sohn, Kyu Hyung Park, Daniel Duck-Jin Hwang

**Affiliations:** 1 Department of Applied Artificial Intelligence, Sungkyunkwan University, Seoul, Korea; 2 Department of Medicine, Kangwon National University Hospital, Kangwon National University School of Medicine, Chuncheon, Gangwon-do South Korea; 3 Seoul Plus Eye Clinic, Seoul, Korea; 4 Kong Eye Center, Seoul, Korea; 5 Department of Ophthalmology, Hangil Eye Hospital, #35 Bupyeong-daero, Bupyeong-gu, Incheon, 21388 Korea; 6 Department of Ophthalmology, Seoul National University Bundang Hospital, Seongnam, Korea; 7 Catholic Kwandong University College of Medicine, Incheon, Korea

**Keywords:** Eye diseases, Health care, Medical research, Engineering

## Abstract

Central serous chorioretinopathy (CSC) is a common condition characterized by serous detachment of the neurosensory retina at the posterior pole. We built a deep learning system model to diagnose CSC, and distinguish chronic from acute CSC using spectral domain optical coherence tomography (SD-OCT) images. Data from SD-OCT images of patients with CSC and a control group were analyzed with a convolutional neural network. Sensitivity, specificity, accuracy, and area under the receiver operating characteristic curve (AUROC) were used to evaluate the model. For CSC diagnosis, our model showed an accuracy, sensitivity, and specificity of 93.8%, 90.0%, and 99.1%, respectively; AUROC was 98.9% (95% CI, 0.983–0.995); and its diagnostic performance was comparable with VGG-16, Resnet-50, and the diagnoses of five different ophthalmologists. For distinguishing chronic from acute cases, the accuracy, sensitivity, and specificity were 97.6%, 100.0%, and 92.6%, respectively; AUROC was 99.4% (95% CI, 0.985–1.000); performance was better than VGG-16 and Resnet-50, and was as good as the ophthalmologists. Our model performed well when diagnosing CSC and yielded highly accurate results when distinguishing between acute and chronic cases. Thus, automated deep learning system algorithms could play a role independent of human experts in the diagnosis of CSC.

## Introduction

Central serous chorioretinopathy (CSC), an eye disease characterized by a serous detachment of the neurosensory retina at the posterior pole^1,2^, is the fourth most common retinopathy following age-related macular degeneration (AMD), diabetic retinopathy, and branch retinal vein occlusion^3,4^. The majority of patients are men with decreased and/or distorted vision together with altered color sensitivity^5^, and CSC is associated with a decrease in the patient's quality of life^6,7^. Multimodal imaging is important for accurately diagnosing CSC. Using multiple modalities such as fluorescein angiography (FA), indocyanine green angiography (ICGA), optical coherence tomography (OCT), and fundus autofluorescence (AF) can help practitioners to distinguish CSC from other retinal diseases with similar clinical features^2^. Among these modalities, OCT is noninvasive and does not require contact, and is often used to evaluate the structural abnormalities associated with CSC. Previous studies have used OCT to investigate the detailed changes in retinal pigment epithelium (RPE) and outer retina morphology^2,8–11^. Additionally, OCT can both assess and quantify the presence of subretinal fluid (SRF), which can facilitate estimation of the episode duration and help determine the subsequent treatment strategy^2^.

Although there has been much effort applied to assess CSC using OCT^12–17^, to the best of our knowledge, no study has reported the use of deep learning techniques to distinguish acute CSC from chronic CSC. When diagnosing CSC, it is important to evaluate the chronicity of the disease to either determine the treatment plan or predict the prognosis. Acute CSC usually has a self-limited natural course, whereas chronic CSC with/without sustained sensory retinal detachment (SRD) may already involve irreversible poor vision or need active intervention, such as focal laser treatment, intravitreal anti-vascular endothelial growth factor injections, or photodynamic therapy; all of these are aimed at preventing permanent visual disturbance that can reduce the patient's quality of life.

Here, we propose and evaluate a deep learning systems (DLS) model for diagnosing CSC and its chronicity using OCT images. Recent advances in DLS techniques such as convolutional neural networks (CNNs) have provided an alternative method to characterize medical image data^18–20^. In ophthalmology, previous studies have reported the high accuracies possible when using CNN-based models for the detection of CSC from fundus photographs, detection and classification of diabetic retinopathy from fundus photographs, detection of AMD from fundus photographs or OCT, visual field examination of glaucoma patients, and the grading of pediatric nuclear cataracts^4,20–25^. In this study, we propose a DLS model that uses OCT scans to distinguish between eyes with CSC and normal healthy eyes. In addition, the model distinguishes between acute and chronic CSC.

## Results

A total of 2,360 images from the 220 participants were included in the study. The mean age of the participants in the normal group was 43.32 ± 13.68 years and that in the CSC group was 46.92 ± 9.54 years. Men constituted 79.03% and 79.75% of the participants in the normal and CSC groups, respectively. Detailed information of the data used in this study is shown in Table 1.

**Table 1:** Baseline characteristics of patients who had undergone macular OCT.

	Normal	CSC		
		Acute CSC	Chronic CSC	Total
Image, no	900	466	994	1460
Patients, no	62	52	106	158
Age(yrs), mean (SD)	43.32 (13.68)	44.87 (8.48)	48.06 (9.94)	46.92 (9.54)
**Gender, no (%)**				
Male	49 (79.03)	40 (76.92)	86 (81.13)	126 (79.75)
Female	13 (20.97)	12 (23.08)	20 (18.87)	32 (20.25)
**Eye, no. (%)**				
Right	34 (54.84)	22 (42.31)	57 (53.77)	79 (50)
Left	28 (45.16)	30 (57.69)	49 (46.23)	79 (50)

OCT, optical coherence tomography; CSC, central serous chorioretinopathy; SD, standard deviation.

### Model performance

**Figure 1:**
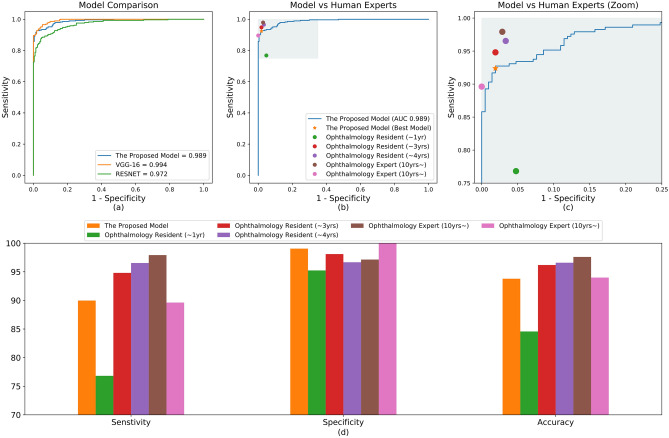
Performances of the model in classifying CSC and normal eyes. (**a**) ROC curve comparison with two popular CNN-based architectures: VGG-16 and Resnet-50. The AUROC of our model was 0.989%, which is slightly lower than VGG-16 and higher than Resnet-50. (**b**) Performance comparison between our model and ophthalmologists. The blue line (ROC curve) is created by sweeping a threshold over the predicted probability for a specific clinical diagnosis. The asterisk denotes our model’s performance with the optimal threshold. (**c**) An expanded version of (**b**). (**d**) Sensitivity, specificity, and accuracy of our model and five ophthalmologists including two human experts. The accuracy is the number of true positives and the number of true negatives divided by the total number of test images. AUROC, area under the receiver operating characteristic curve; CNN, convolutional neural network; CSC, central serous chorioretinopathy; ROC, receiver operating characteristic.

**Figure 2:**
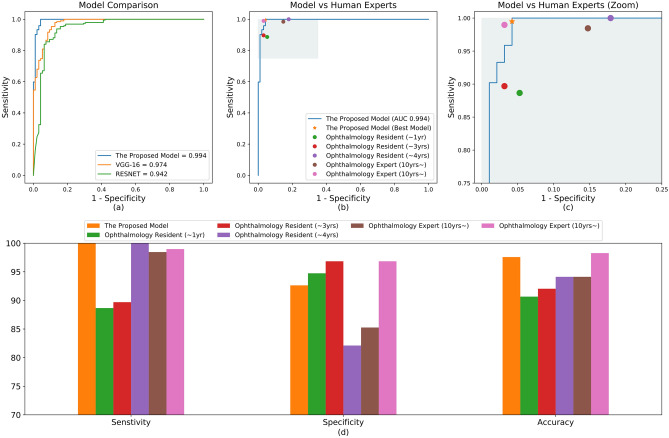
Performances for acute CSC vs chronic CSC classification. (**a**) ROC curve comparison with two popular CNN-based architectures: VGG-16 and Resnet-50. The AUROC of our model was 0.994%, which outperforms both VGG-16 and Resnet-50. (**b**) Performance comparison between our model and ophthalmologists. The blue line (ROC curve) is created by sweeping a threshold over the predicted probability for a specific clinical diagnosis. The asterisk denotes our model’s performance with the optimal threshold. (**c**) An expanded version of (**b**). (**d**) Sensitivity, specificity, and accuracy of our model and five ophthalmologists. Our model’s performance is better than most of the ophthalmologists. AUROC, area under the receiver operating characteristic curve; CNN, convolutional neural network; CSC, central serous chorioretinopathy; ROC, receiver operating characteristic.

The results from the proposed model are shown in Table 2. There were 29 cases in which our model incorrectly judged CSC as normal; Table 3 shows six representative examples. Of these 29 cases, three were acute CSC with SRF and the remaining 26 cases were chronic CSC without SRF. The AUROC (Fig. 1), was 98.9% (95% confidence interval [CI], 0.983–0.995), which was less than that of VGG-16 (99.4%) but better than that of Resnet-50 (97.2%). The AUROC of the model for distinguishing chronic from acute CSC was 99.4% (95% CI, 0.985–1.000), which was better than that of both VGG-16 (97.4%) and Resnet-50 (94.2%) (Fig. 2).

**Table 2:** The sensitivity, specificity, and accuracy of the model for diagnosing CSC and classifying acute and chronic CSC OCT images.

	Normal versus CSC^a^	
	Predicted normal	Predicted CSC
Actual Normal	208	2
Actual CSC^a^	29	260

	Acute CSC versus chronic CSC^b^	
	Predicted acute CSC	Predicted chronic CSC
Actual Acute CSC	88	7
Actual Chronic CSC^b^	0	194

CSC, central serous chorioretinopathy; OCT, optical coherence tomography.

^a^The sensitivity of the classifier for detecting CSC eyes was 90.0%, the specificity was 99.1%, and the accuracy was 93.8%.

^b^The sensitivity of the classifier for detecting chronic CSC was 100.0%, the specificity was 92.6%, and the accuracy was 97.6%.

### Performance comparison with ophthalmologists

The performances of the ophthalmologists are shown in Figs. 1 and 2. Their classification accuracies for diagnosing CSC were between 84.6%–97.6%, and for distinguishing chronic from acute CSC were 90.7%–98.3%, whereas our model had accuracies of 93.8% and 97.6%, respectively. Therefore, our model’s performance was comparable in diagnosing CSC, and comparable or better in distinguishing acute from chronic CSC with the performance of the ophthalmologists. The Kappa coefficients for the two human experts was 0.855 (*P* < 0.001) in diagnosing CSC, and 0.887 (*P* < 0.001) in distinguishing chronic from acute CSC, showing good agreement. There were 11 cases of inconsistency in which the two experts differed in distinguishing chronic from acute CSC cases, which our model classified correctly.

### Grad class activation mapping

**Figure 3:**
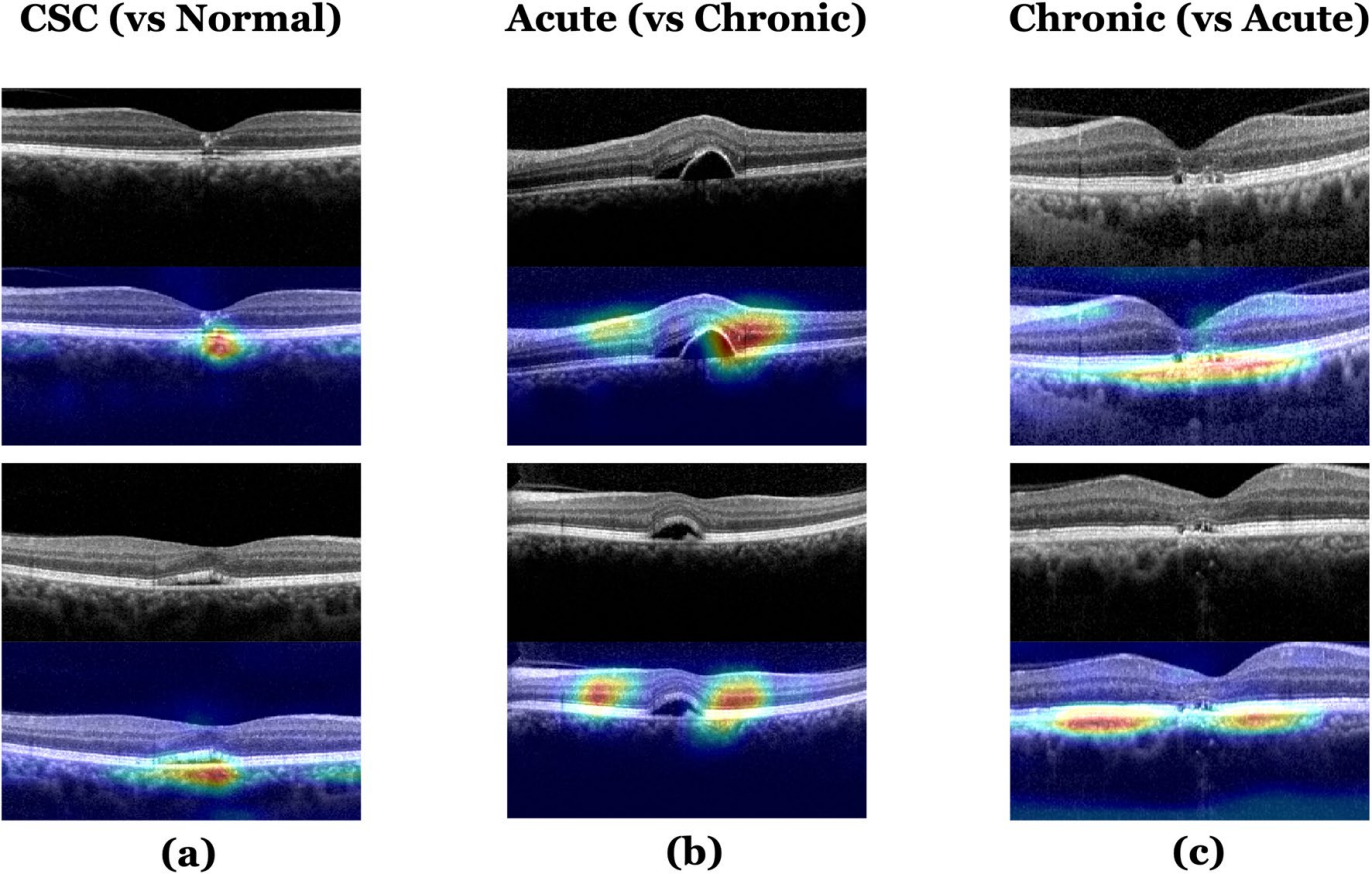
Heat maps for the classification models by Grad-CAM. (**a**) A heat map for the model classifying normal and CSC OCT images; (**b**), (**c**) Heat maps for the model classifying acute and chronic CSC. The Grad-CAM was able to identify pathologic regions on the OCT, which are presented as a heat map. CSC, central serous chorioretinopathy; Grad-CAM, gradient weighted class activation mapping; OCT, optical coherence tomography.

Representative heat maps produced from the two classifications by the Grad-CAMs are shown in Fig. 3. The heat maps highlighted regions that were comparable with the region that retina specialists usually consider when diagnosing CSC. This shows that our model uses a similar approach in assessing CSC images.

## Discussion

In this study, we built a DLS model and investigated its performance in using SD-OCT images to diagnose CSC and distinguish chronic CSC from acute CSC. Several studies^12–17^ have previously analyzed CSC using OCT, however most of these were for segmentation, rarely for classification (Table 4). Our model showed promising performance in diagnosing CSC and performed well in distinguishing between acute and chronic CSC, where its performance was either comparable to, or better than, experienced retina doctors. This study is the first to evaluate the performance of a DLS model that classifies acute and chronic CSC using OCT.

**Table 3:**
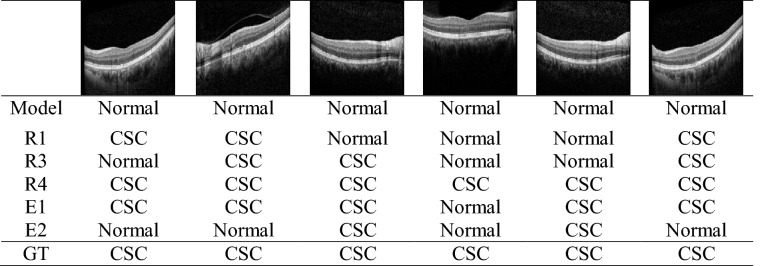
Examples of decisions made by the model and five human experts.

The six images were false negatives detected by our model in normal versus CSC classification.

CSC, central serous chorioretinopathy.

R1, R3 and R4 denote ophthalmology residents with less than 1 year, 3 years, and 4 years of experience, respectively. E1 and E2 refers to ophthalmology experts with more than 10 years of experience. GT denotes the ground truth.

**Table 4:** Summary table for describing prior studies that applied the machine learning methods in assessing CSC using OCT images.

Authors	Year	OCT machine	Test images	Task	Model	Remarks
Ruan et al^14^	2019	Cirrus SD-OCT	1280	Retinal layer segmentation	CNN FCN-MLS	Deep learning
Xiang et al^15^	2019	Cirrus HD-OCT 4000	48	Retinal layer segmentation	Random forest classifier	Feature engineering
Gao et al^16^	2019	unknown	52 OCT volumes	Subretinal fluid segmentation	CNN DA-FCN	Deep learning
Novosel et al^17^	2016	Spectralis OCT	10	Retinal layer and fluid Segmentation	LCLS	Probabilistic framework
Syed et al^12^	2016	Topcon 3D TD-OCT	90 OCT volumes	Classification CSC versus ME versus Normal	SVM	Feature engineering
Khalid et al^13^	2017	Topcon 3D OCT-2000	2819	Classification CSC versus ME versus ARMD versus Normal	SVM	Feature engineering
Our study	2020	Spectralis OCT	788	Classification CSC versus normal and acute CSC versus chronic CSC	CNN Custom deep neural network using 13 CNN layers and 4 FC layers	Deep learning Interpret the results with Grad-CAM

CSC, central serous chorioretinopathy; OCT, optical coherence tomography; FCN-MLS, fully convolutional network + multiphase level set; DA-FCN, double-branched and area constraint fully convolutional network; LCLS, locally-adaptive loosely coupled level set; SVM, support vector machine; CNN, convolutional neural network; FC, fully connected.

There were 29 false negatives where the model incorrectly classified CSC images as normal, which resulted in a relatively low sensitivity (90%). This could be due to the following reasons. First, there is no universally accepted classification system for CSC, nor is there a consensus on what constitutes chronic CSC^26,27^. In this study, chronic CSC without SRD was included in the chronic CSC group, and some chronic CSC images without SRF could be confused as normal. Twenty-six out of the 29 false negatives were chronic CSC, and none of these 26 cases had SRF on the images. If we had only considered CSC cases with SRF when training and testing the model, it is likely that there would have been fewer false negatives, which would consequently have improved the performance of our model. Second, we used randomly selected non-centered image cuts as well as five centered image cuts showing the typical CSC pattern. Therefore, even if the centered image cuts of an OCT volume revealed prominent acute or chronic CSC characteristics, the non-centered image cuts may have shown similar characteristics to normal eyes, resulting in the misjudgment. Interestingly, all except four of the false negative cases were non-centered images.

From a clinical perspective, it is important that a classification model shows a high sensitivity by reducing false negatives. If a normal case is incorrectly classified as CSC, i.e. a false positive, it may increase the burden on the healthcare system. However, if a CSC case is incorrectly classified as normal, i.e. a false negative, it can cause a serious problem for the patient; irreversible visual impairment and visual function deterioration may occur if the appropriate treatment is not initiated quickly. Therefore, we plan to improve the performance (especially the sensitivity) of our model on SRF-free and non-centered OCT images, even though the accuracy of our current model was comparable to that of the ophthalmologists.

Our model had a better sensitivity and accuracy than most of the ophthalmologists in distinguishing between acute and chronic CSC, and correctly classified 11 cases that two ophthalmology experts differed on. This implies that the model can provide useful information for diagnosis even when human experts with good agreement give differing interpretations. Therefore, our model performed promisingly in distinguishing chronic from acute CSC, and demonstrated a unique potential for using DLS technology to assess CSC based on OCT.

Improvements in OCT technology have increased the number of OCT images that are generated, subsequently increasing the amount of OCT data to be analyzed and pushing the limits of clinical capacity. Therefore, image analysis using DLS is expected to contribute increasingly in the future. CNNs are popular neural networks with many layers that perform particularly well in image recognition^19^. Our CNN included an iterative convolutional layer structure responsible for extracting local features of the image, and a pooling layer that summarized the features of each region. Unlike conventional machine learning classifiers, the CNN can use these automatically extracted features to accurately classify an image. By applying a CNN model to OCT images, both classification and segmentation can be performed. In classification, the model predicts the class for an unlabeled image, whereas in segmentation, the model tries to predict the class of a pixel in an image, and not the entire image. Hence, the final output of the model in the segmentation task can be an image comprising a set of labeled (or classified) pixels. In this study, we conducted two binary classification tasks. To feed our DLS CNN, we preprocessed the OCT images in two steps: cropping and resizing. We cropped the original OCT images to remove unnecessary parts, and then resized the cropped image to an input size of 224 × 224 pixels for our model, which is widely used in CNN architectures. To build a robust model applicable to a variety of input images, we performed an image augmentation process in the training phase. When we initially trained the model without data augmentation, we found that in some instances images that were tilted were misclassified. To address this issue, we randomly rotated, changed the brightness, and horizontally flipped the images, and subsequently only included augmented images in the training phase. This augmentation notably improved the performance of our model, confirming that the preprocessing methods that we used were effective, especially when dealing with a relatively small data set.

We showed that the Grad-CAM can correctly identify the pathologic region of an OCT image. The purpose of using Grad-CAM is to identify and specify the parts of an image that affect the probability scores of each class. The heat map of the regions activated by the model can identify and quantify differences, highlighting the important areas in the classification process. In clinical practice, we often observe CSC cases where the time alone cannot fully explain the chronicity of the disease. For example, some patients possess acute CSC findings even though their symptoms are more than 1 year old, while others have chronic CSC findings with extensive atrophic changes in the macula, despite their symptoms being less than 1 month old. Therefore, evaluating CSC chronicity with OCT as a biomarker alongside the time (actual onset time or time when the patient's symptoms were present), is more reliable than judging by the time alone.

In our study, we only used OCT images without information on the time variables in the proposed model. The highlighted part in the heatmap generated from GRAD-CAM refers to the location that is activated when the model classified a specific class (i.e., normal vs. CSC or acute CSC vs. chronic CSC). Such heatmaps are often beneficial when interpreting what the model considers for decision making. As shown in Fig. 3, the highlighted area on the heatmap presents all the inner retina, outer retina, and choroid layer, which includes the location of several known OCT biomarkers, including alteration of the RPE and outer retina morphology^2,8–11^. Hence, we believe that using Grad-CAM during the learning process with OCT images (without any information on time) can provide useful detailed biomarkers for evaluating chronicity. Additionally, where many OCT images obtained by frequent examinations with long-term follow-up require analysis, the Grad-CAM result could be used to shorten the analysis time, avoid oversight, and help ophthalmologists arrive at a consistent prognosis.

This study has some limitations. First, the variety and number of OCT images available were limited. External validation is necessary in future studies because all the images in this study were acquired from a single institution. However, the dataset was sufficient to demonstrate the feasibility of our DLS model to diagnose CSC and distinguish chronic from acute CSC using OCT images. Second, the model could be extended toward predicting future disease progression through a series of OCT images. In addition to determining the current status by viewing the latest image, the extended model could predict the future progression or chronicity using the longitudinal image data of CSC patients. Recurrent neural networks and long-short term memory can be used in such sequential predictions. Third, since we did not find any similar investigations using OCT images to classify CSC from normal retinas and chronic from acute CSC, we could not compare performance with previous studies. Regardless of the above limitations, the developed model demonstrates a reasonable and promising performance and suggests the need for further investigations on its potential impact in clinical practice.

In our study, we developed and evaluated a deep learning model that can diagnose CSC and distinguish its chronicity using SD-OCT images, which can be clinically useful in either determining the treatment plan or predicting prognosis. In clinical practice, a patient may present several macular diseases (e.g., CSC, AMD, etc.) at the same time. In addition, even if only one macular disease is present, the patient may potentially possess several other typical macular diseases such as AMD, DR, and RVO in addition to CSC. Therefore, developing a model that can identify each macular disease and its severity is crucial from a clinical perspective. Such a model can be useful in simultaneously assessing the presence or absence of several macular diseases, and help with the correct diagnosis.

In summary, we developed a DLS CNN model that performed well at diagnosing CSC and distinguishing chronic CSC from acute CSC without a segmentation algorithm. The process for assessing CSC needs to maximize its capacity to process the increasing number of images from participants who have examinations, with high accuracy. Automation of the classification process using DLS models may improve patients' quality of life by improving prognosis and may save cost and time for both healthy people and patients with CSC.

## Methods

This study was conducted in line with the Helsinki Declaration of 1964. The Ethics Committee of Hangil Eye Hospital approved the research protocols and their implementation. The committee waived the requirement for obtaining informed consent given that this was a retrospective observational study of medical records and was retrospectively registered.

### Data collection and labelling

We analyzed the records of patients who visited Hangil Eye Hospital between January 2017 and January 2020. We used spectral domain (SD)-OCT (Heidelberg Spectralis, Heidelberg Engineering, Heidelberg, Germany) images of normal participants, and of patients with CSC. Of the 220 patients enrolled at the outpatients’ clinic during that period, 158 were diagnosed with CSC and 62 were normal healthy patients who were assigned to the control group. All CSC cases were diagnosed by means of fundus examinations, FA, ICGA, and OCT images by independent retinal specialists. A confocal scanning laser ophthalmoscope (Heidelberg Retina Angiograph, HRA; Heidelberg Engineering, Heidelberg, Germany) was used to perform simultaneous FA and ICGA on all CSC cases. One eye per patient was selected for this study, with one visit per patient. Our analysis excluded data that showed the presence of other potentially conflicting retinal pathologies such as AMD, polypoidal choroidal vasculopathy, pachychoroid neovasculopathy, and pachychoroid pigment epitheliopathy. We randomly selected 5–10 non-centered image cuts from the 25 volume scan image cuts for each OCT volume, as well as five centered image cuts showing the typical CSC pattern.

Acute CSC: Acute CSC was diagnosed based on the presence of serous detachment of the neurosensory retina involving the macula as demonstrated by OCT, and leakage at the level of the RPE on FA. Only classic, acute CSC with a symptom duration of less than 3 months since the first episode, was included in the acute CSC group.

Chronic CSC: Based on the Daruich and colleagues’ classification scheme^27^, chronic CSC was diagnosed according to the RPE status and was defined as chronic chorioretinopathy with widespread RPE decompensation, with/without SRD, and with/without an active leakage site. As their definition, chronic CSC was diagnosed when extensive RPE atrophy findings were observed regardless of SRF.

Categorization: Categorization was performed by two retina specialists (JSH and DDH) who examined all images obtained by OCT, FA, and ICGA multimodal imaging methods, and reviewed the medical charts. In cases of disagreement, a third retina specialist (JMH) confirmed the discrepancy and discussed the case with the other specialists. After a discussion, all discrepancies were resolved by consensus.

### Data preprocessing

To use the SD-OCT images as input for a DLS CNN, we first removed the unnecessary parts (such as the company logo) from the original 596 × 1264 pixel SD-OCT images, which resulted in 380 × 764 pixel RGB images. We subsequently down-sampled the 380 × 764 pixel cropped images to 224 × 224 pixel RGB images, which were fed into the DLS CNN. Images of 224 × 224 pixel RGB are a widely-used image standard for classification models such as VGG-16^28^ and Resnet-50^29^. To avoid overfitting^30^, we performed a data augmentation process to build a robust model from a variety of input images. The data augmentation process included random horizontal image flips, random brightness changes from 0.7 to 1.3, and random rotations of the image of up to 15^°^. This data augmentation process was only applied in the training phase.

### Model architecture

**Figure 4:**
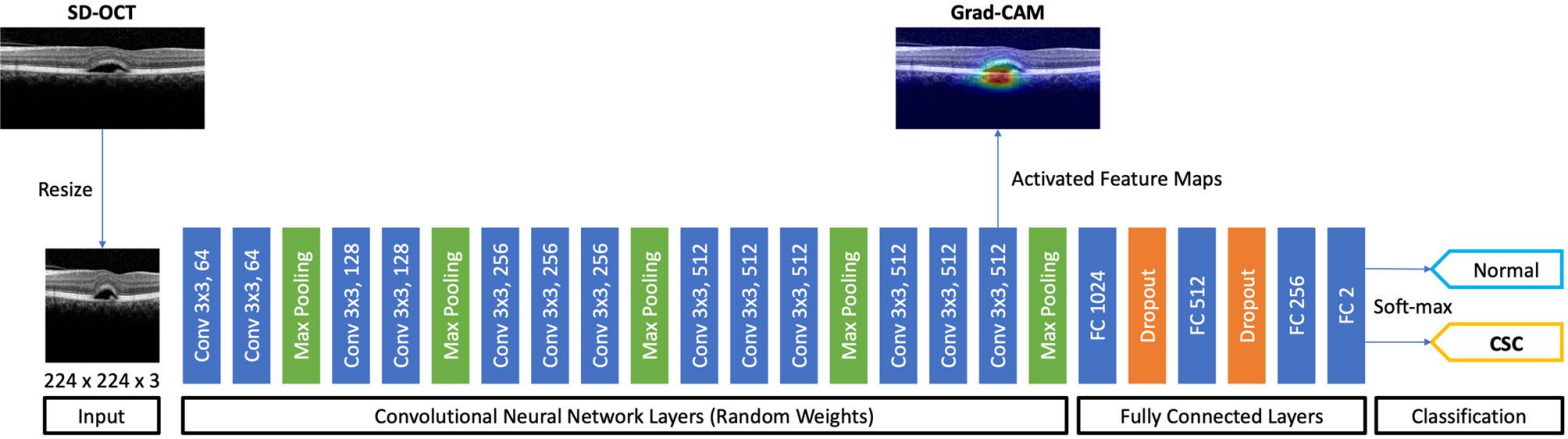
An illustration of the proposed model used in classifying CSC and normal eyes. Our model comprises an input layer, 13 CNN layers with ReLU activation functions, 4 max pooling layers, 2 dropout layers, and 4 FC layers. The last FC layer was used for binary classification. The heat map was generated from the final CNN layer. CNN, convolutional neural network; CSC, central serous chorioretinopathy; FC, fully connected; Grad-CAM, gradient weighted class activation mapping ReLU, rectified linear unit; SD-OCT, spectral domain optical coherence tomography.

To classify a given OCT image as either CSC or normal, we built a DLS model based on the CNN architecture. As shown in Fig. 4, the proposed model comprised 13 CNN layers with a rectified linear unit (ReLU) activation function^31^, four max pooling layers, two dropout layers, and four fully connected (FC) layers. The dropout layer helped our model to avoid overfitting^28^, and the FC layers formed a traditional multilayered perceptron^32^. The final output layer with a soft-max activation function was used to predict the binary classification result. The proposed CNN used 118,132,802 trainable parameters. The proposed architecture was also used to classify whether a CSC OCT image was either acute or chronic; the last output layer with the soft-max activation function in the model was replaced for the binary classification between acute and chronic CSCs. We considered applying the transfer learning method to our model as other researchers have done^33,34^; however, we decided not to use it because it performed poorly on our dataset.

### Gradient weighted class activation mapping

To visualize the pathologic region of an OCT image in the classification process, we applied gradient weighted class activation mapping (Grad-CAM)^35^ to generate a heat map of activated regions. Grad-CAM uses the gradients of the target label (e.g., CSC) with respect to feature maps of the convolutional layer to highlight important regions in the image when predicting the target label. The heatmap illustrates the area of the image that the model uses for its classification.

### Experiment setup

The data were randomly split into a training (1,861) and test set (499). The test set was used only for the final evaluation of the model performance; no single patient case existed in both sets. To diagnose CSC, the training and test sets were split CSC/normal 1,171/690, and 289/210, respectively. To classify the CSC cases the acute/chronic split of the training and test sets were 371/800, and 95/194, respectively.

To compare our model’s performance with DLS architectures reported previously, including VGG-16^28^ (VGG with 16 layers) and Resnet-50^29^ (Resnet with 50 layers), we used our training and test sets on these models. All models were trained with a batch size of 64, epochs of 50, and with Adam optimization (learning rate 0.0001). To evaluate our model from a clinical perspective, the classification results for the test set (788 images) were compared with those made by five ophthalmologists, including three ophthalmology residents and two experts, each having more than 10 years clinical experience at an academic ophthalmology center.

### Statistical analysis

To measure the performance of the model, the sensitivity, specificity, accuracy, and the area under the receiver operating characteristic (ROC) curve (AUROC)^36^ were determined. Cohen’s Kappa coefficients were used to rate the agreement level between the two experts.

## Data Availability

The data are not available for public access because of patient privacy concerns, but are available from the corresponding author on reasonable request.
